# The prevalence of impaired fasting glucose and undiagnosed diabetes mellitus and associated risk factors among adults living in a rural Koladiba town, northwest Ethiopia

**DOI:** 10.1186/s13104-017-2571-3

**Published:** 2017-07-06

**Authors:** Abebaw Worede, Shitaye Alemu, Yalemzewod Assefa Gelaw, Molla Abebe

**Affiliations:** 1 University of Gondar Teaching Hospital Laboratory, Gondar, Ethiopia; 2 Department of Internal Medicine, School of Medicine, College of Medicine and Health Sciences, University of Gondar, Gondar, Ethiopia; 3 Department of Epidemiology and Biostatistics, Institute of Public Health, College of Medicine and Health Sciences, University of Gondar, Gondar, Ethiopia; 4 Department of Clinical Chemistry, School of Biomedical and Laboratory Sciences, College of Medicine and Health Sciences, University of Gondar, Gondar, Ethiopia

**Keywords:** Impaired fasting glucose, Undiagnosed diabetes mellitus, Risk factor, Ethiopia

## Abstract

**Background:**

Diabetes mellitus is becoming a big public health challenge, particularly in developing countries like Ethiopia. It is a manageable disease if early screening and follow up is made. However, as studies in Ethiopia are limited and unorganized, determining the magnitude of prediabetes and diabetes and identifying associated risk factors is quite essential.

**Methods:**

A community-based, cross-sectional study was conducted from February to April 2015 among adults (aged ≥20 years) in a rural Koladiba town. A multistage sampling technique was used to select a total of 392 study participants. Data were collected after a fully informed written consent was obtained from each participant. Demographic, behavioral, and clinical data were collected using a well-structured questionnaire. Multivariable logistic regression models were fitted to control the effect of confounders. Adjusted odds ratios (AOR) with their 95% confidence intervals (95% CI) were computed to measure associations. A p value of <0.05 was considered as statistically significant.

**Results:**

The prevalence of impaired fasting glucose and undiagnosed diabetes mellitus were 12% (95% CI 9–16) and 2.3% (95% CI 1.1–4), respectively, in Koladiba. Overweight (AOR: 4.257, 95% CI 1.345–13.476), obesity (AOR: 5.26, 95% CI 1.138–24.316), hypertriglyceridemia (AOR: 2.83, 95% CI 1.451–5.521), and systolic hypertension (AOR: 3.858, 95% CI 1.62–9.189) were found to be independently associated with impaired fasting glucose. Positive family history of diabetes also showed a marginal association with impaired fasting glucose (p = 0.057). Male sex (p = 0.012) and hypertriglyceridemia (p = 0.030) were associated with undiagnosed diabetes mellitus.

**Conclusions:**

The prevalence of impaired fasting glucose and undiagnosed diabetes mellitus are found to be significant. Obesity, hypertriglyceridemia, and systolic hypertension are independently associated with impaired fasting glucose among adults. We recommend that the community be aware of healthy life style, early screening, and maintain continuous follow up.

## Background

Diabetes mellitus (DM) is a metabolic disorder of multiple etiologies, characterized by chronic hyperglycemia with disturbances of carbohydrate, fat, and protein metabolism resulting from defects in insulin secretion, action or both [1]. Diabetes is one of the most common metabolic disorders in the world, and its prevalence has been increasing in the last decades [2]. Diabetes exerts a high burden in sub-Saharan Africa, and this is expected to increase from time to time. Many DM patients face challenges in accessing diagnosis and treatment in this region which contributes to significant morbidity and mortality rates [3].

Ethiopia is one of the four most populous countries which have the highest numbers of people with diabetes in Africa. The number of people with diabetes is increasing in every region of Ethiopia. According to International Diabetes Federation Atlas (IDFA) in 2013, there were 1.9 million diabetes cases of 20–79 years aged people and 34,262 diabetes related deaths and 1.2 million of them were in the rural setting. More than 1 million of this people were living with undiagnosed diabetes mellitus [4]. The estimated prevalence of diabetes among the Ethiopian adult (20–79 years) population was 2% [5], 3.5% [2] and 4.4% [6] in 2010, 2011 and 2013, respectively. Increasing urbanization, aging population, obesity, and physical inactivity are all contributing to the rise of diabetes worldwide [7].

Individuals with impaired fasting glucose (IFG) have been referred to as having prediabetes, indicating the relatively high risk for the future development of diabetes. IFG should not be viewed as a clinical entity in its own right but rather as a risk factor for diabetes and cardiovascular disease (CVD). It is associated with obesity, dyslipidemia, and hypertension [8, 9].

The chronic hyperglycemia of DM is associated with a long-term damage, dysfunction, and failure of various organs, especially the eyes, nerves, kidneys, heart, and blood vessels [10]. As a result of poor diagnosis and awareness in communities, DM complications are common in sub-Saharan Africa. Thus, people with diabetes (known or unknown) visit health facilities because of complications rather than for routine consultation or follow-up [11]. DM is a chronic illness that requires ongoing medical care and continuing patient self-management education and support to prevent acute complications and to reduce the risk of long-term complications. In addition, diabetes care is complex and needs multifactorial risk reduction strategies beyond glycemic control [8].

Findings from different studies indicated that individuals with prediabetes could prevent the onset of type 2 diabetes by losing their body weight through physical exercise and dietary management, behavior modification and medications [12, 13]. Although IFG is an early alert system which offers a warning to prevent the future development of DM and diabetes-related complications, there is limited data regarding the prevalence of prediabetes (IFG), undiagnosed DM and associated risk factors in Ethiopia. Therefore, this study will provide baseline information regarding the magnitude of prediabetes, undiagnosed DM and associated risk factors in Ethiopian setting, which will be used to minimize the increasing prevalence of DM and associated complications.

## Methods

### Study population, design, setting and period

The study included adults (20 years and above) who were living in the study area and volunteered to give informed written consent. Pregnant women, individuals who were taking drugs with a possible impact on glucose metabolism (e.g. steroids, B-blockers, thiazide diuretics), diabetes mellitus confirmed individuals, and critically ill patients who were not able to communicate were excluded from the study.

A community-based, cross-sectional study was conducted from February to April 2015 in a rural Koladiba town of Dembia district. Koladiba town is located in the Amhara National Regional State, 729 km from Addis Ababa, the capital city of Ethiopia. The town has 17,500 inhabitants, living in 8 small administrative unites called “Ketenas”, very close to Lake Tana. The town has one health center.

### Sampling techniques and sample size determination

A multistage sampling technique was applied to select the participants. Four ketenas (smallest administrative units with similar demographics) were selected randomly via the lottery method from a total of 8 ketenas. Then, all households in the 4 ketenas with the age of 20 years and above were considered as a sampling unit. A random sampling technique (lottery method) was employed to select each household proportionally (because the number of households in selected ketenas was not equal). When there was more than one eligible subject in each household, one of them was selected randomly by the lottery method. The sample size was determined using the single population proportion formula by considering: a 15% prevalence of prediabetes from a previous study in southwest Ethiopia [14], a 5% level of significance, and a 0.05% margin of error. The calculated result (196) was multiplied by the design effect of 2; therefore, the final sample size was 392.

### Data collection

Demographic, behavioral, and clinical data of the study participants were collected using a pretested and structured questionnaire. Data collectors were trained nurses supervised by experienced professionals. Blood sample was also collected by trained medical laboratory professionals. A 4-day training and practical demonstration was given to the data collectors on the objective of the study, procedure of blood sample collection, procedures of measurement, confidentiality of information, respondents’ rights and the techniques of interview prior to the data collection.

After the interview was made in the evening, the participants were instructed to be on overnight fasting (10–16 h). The next morning, 3–5 ml venous blood was collected using a plain vacutainer tube. The blood sample was left at room temperature to be clotted for 15–20 min and centrifuged at 3000 rpm for 10 min. Then, sera was transferred to 2 ml Eppendorf tubes and stored at 4 °C for 1–2 h at sample collection sites and transported to the Clinical Chemistry Laboratory of the University of Gondar Hospital. The blood level of glucose was determined by the glucose oxidase method and the total cholesterol and triglycerides were measured by the enzymatic colorimetric method using the Mindray BS-200 Chemistry Analyzer (Mindray Medical International LTD, China).

Normal fasting blood glucose (FBG) refers to FBG level below 100 mg/dl, without a history of diabetic medication. Impaired fasting glucose (IFG) refers to a level of blood glucose between 100 and 125 mg/dl with no diabetic medication. Diabetes manifests when the FBG level equals or exceeds 126 mg/dl, or a history of diabetic medication exists. Undiagnosed diabetes mellitus is defined as unknowingly having an elevated glucose level that meets the definition of diabetes mellitus [10]. The cutoff points to categorize hyperlipidemia were total cholesterol (TC) ≥200 mg/dl and triglycerides (TG) ≥150 mg/dl [15].

### Measurements

Standardized techniques and calibrated equipments were used for anthropometric measurements. Each eligible subject was weighed to the nearest 0.1 kg in light indoor clothing and bare feet or with stockings. Body mass index (BMI) (kg/m^2^) was calculated using study participants’ height and weight. Height was measured using a portable stadiometer. BMI was classified as <18.5 kg/m^2^ underweight, 18.5–24.9 kg/m^2^ normal, 25–29.9 kg/m^2^ overweight and ≥30 kg/m^2^ obese. Waist circumference (WC) was measured at the midpoint between the lower margin of the least palpable rib and the top of the iliac crest. WC values of >102 and >88 cm for males and females, respectively, were considered high, according to World Health Organization.

The systolic blood pressure (SBP) and diastolic blood pressure (DBP) of each participant were measured two times using a mercury sphygmomanometer, while the participants were in a sitting position and after 15 min’ rest. The second BP measurement was taken after 5 min of the first measurement. Then, the average value was calculated and taken as the BP result. SBP ≥ 140 mmHg or DBP ≥ 90 mmHg or reported use of regular anti-hypertensive drugs were considered to define hypertension [15].

### Data analysis

The statistical package for social sciences (SPSS) version 20 (IBM, USA) software was used for data entry and analysis. Bivariable and multivariable logistic regression models were fitted to identify possible associated risk factors with impaired fasting glucose. Variables with a p value of ≤0.2 in the bivariable analysis were maintained in the multivariable model to control the effect of confounding variables. The Hosmer–Lemeshow goodness-of-fit statistic was used to assess the fitness of the model. Both crude and adjusted odds ratios with their 95% confidence intervals (CI) were computed to measure the strengths of associations between variables. However, odds ratios can be biased when the rare disease assumption is violated, generally when the prevalence is greater than 10%, which is the case for IFG in this population. Fisher’s exact test was used to evaluate the association of risk factors with undiagnosed diabetes mellitus. A p value of <0.05 was considered as statistically significant.

### Operational definitions

Family history of DM was considered as positive if either or both parents or siblings of individuals were diagnosed to have DM. Smoking habit was reported as “nonsmoker” (for individuals who never smoked) and “smoker” (for individuals who were smoking at the moment).

## Results

### Socio-demographic characteristics of study participants

Five percent of the selected individuals were disagreed to participate in this study because of fear of venipuncture for blood sample collection and other reasons. Additional participants were invited in place of the above disagreed individuals to fulfill the calculated sample size. Three hundred ninety-two adult participants with the mean age of 43.76 ± 17.29 years (range 20–90 years) were included. The majority, 219 (55.9%), of the participants were females; 235 (59.9%) were married; 336 (85.7%) were Orthodox christian and 126 (32.1%) unable to read and write (illiterate) (Table 1).

**Table 1:** Socio-demographic characteristics of study participants in Koladiba town of Dembia district, northwest Ethiopia, 2015 (n = 392)

Variable	Category	Number	Percent
Sex	Male	173	44.1
	Female	219	55.9
Age	20–29	106	27.0
	30–39	72	18.4
	40–49	67	17.1
	50–59	58	14.8
	>60	89	22.7
Occupation	Employed	85	21.7
	Housewife	114	29.1
	Student	25	6.4
	Merchant	63	16.1
	Daily laborer	75	19.1
	Farmer	30	7.7
Marital status	Married	235	59.9
	Single	60	15.3
	Divorced	45	11.5
	Widowed	47	12.0
	Separated	5	1.3
Educational status	Not read and write	126	32.1
	Primary school	111	28.3
	Secondary school	115	29.3
	Higher education	40	10.2
Religion	Orthodox christian	336	85.7
	Protestant christian	4	1.0
	Muslim	52	13.3

### Prevalence of impaired fasting glucose and undiagnosed DM

According to the American Diabetes Association (ADA) fasting criteria, the prevalence of impaired fasting glucose (IFG) and undiagnosed diabetes mellitus (DM) among participants were 12% (95% CI 9–16) and 2.3% (95% CI 1.1–4), respectively. Among study participants, the prevalence of IFG was almost similar for sexes, 5.9% for males and 6.1% for females (Table 2).

**Table 2:** IFG and undiagnosed DM by socio-demographic characteristics of the study participants in Koladiba town of Dembia district, northwest Ethiopia, 2015

Parameter	NFG (n = 336, % = 85.7) N %	IFG (n = 47, % = 12) N %	UDM (n = 9, % = 2.3) N %
Sex			
Male	142 (36.2)	23 (5.9)	8 (2)
Female	194 (49.5)	24 (6.1)	1 (0.3)
Age			
20–29	99 (25.3)	6 (1.5)	1 (0.3)
30–39	66 (16.8)	6 (1.5)	0 (0)
40–49	51 (13)	13 (3.3)	3 (0.8)
50–59	46 (11.7)	10 (2.6)	2 (0.5)
>60	74 (18.9)	12 (3.1)	3 (0.8)
Occupation			
Employed	71 (18.1)	12 (3.1)	2 (0.5)
Housewife	100 (25.5)	13 (3.3)	1 (0.3)
Student	23 (5.9)	2 (0.5)	0 (0)
Merchant	49 (12.5)	9 (2.3)	5 (1.3)
Daily laborer	68 (17.3)	6 (1.5)	1 (0.3)
Farmer	25 (6.4)	5 (1.3)	0 (0)
Marital status			
Married	196 (50)	31 (7.9)	8 (2)
Single	55 (14)	5 (1.3)	0 (0)
Divorced	42 (10.7)	3 (0.8)	0 (0)
Widowed	38 (9.7)	8 (2)	1 (0.3)
Separated	5 (1.3)	0 (0)	0 (0)
Educational status			
Not read and write	112 (28.6)	14 (3.6)	0 (0)
Primary school	93 (23.7)	13 (3.3)	5 (1.3)
Secondary school	96 (24.5)	16 (4.1)	3 (0.8)
Higher education	35 (8.9)	4 (1)	1 (0.3)
Religion			
Orthodox christian	287 (73.2)	43 (11)	6 (1.5)
Protestant christian	3 (0.8)	1 (0.3%)	0 (0)
Muslim	46 (11.7)	3 (0.8%)	3 (0.8)

*IFG* impaired fasting glucose, *N*/*n* number, *NFG* normal fasting glucose, *UDM* undiagnosed diabetes mellitus, *%* percent

### Factors associated with IFG and undiagnosed DM

In the bivariate analysis, age, smoking habit, family history of diabetes mellitus (DM), body mass index (BMI), waist circumference (WC), total cholesterol (TC), triglycerides (TG), systolic blood pressure (SBP), and diastolic blood pressure (DBP) were found to be significantly associated with the prevalence of impaired fasting glucose (Table 3).

**Table 3:** Bivariate and multivariate analysis of factors associated with IFG in Koladiba town of Dembia district, northwest Ethiopia, 2015 (n = 383)

Variable	Category	IFG		COR (95% CI)	AOR (95% CI)
		Yes	No		
Age	20–29	6	99	1	1
	30–39	6	66	1.5 (0.464–4.851)	0.657 (0.17–2.545)
	40–49	13	51	4.206 (1.510–11.718)	2.275 (0.705–7.34)
	50–59	10	46	3.587 (1.229–10.466)	1.671 (0.495–5.638)
	≥60	12	74	2.676 (0.960–7.459)	1.737 (0.559–5.402)
SH	Non-smokers	42	327	1	1
	Smokers	5	9	4.325 (1.384–13.517)	2.647 (0.714–9.809)
FHDM	Yes	14	35	3.648 (1.782–7.469)	2.193 (0.976–4.926)*
	No	33	301	1	1
BMI	Underweight	5	85	1	1
	Normal	21	205	1.741 (0.636–4.770)	1.419 (0.504–3.993)
	Overweight	16	37	7.351 (2.507–21.557)	4.257 (1.345–13.476)**
	Obese	5	9	9.444 (2.289–38.966)	5.26 (1.138–24.316)**
WC	Low risk	25	249	1	1
	High risk	22	87	2.519 (1.351–4.695)	1.132 (0.474–2.703)
TC	Normal	31	279	1	1
	High	16	57	2.526 (1.296–4.923)	1.713 (0.8–3.668)
TG	Normal	21	245	1	
	High	26	91	3.333 (1.787–6.217)	2.83 (1.451–5.521)***
SBP	Non-hypertensive	35	315	1	1
	Hypertensive	12	21	5.143 (2.333–11.338)	3.858 (1.62–9.189)***
DBP	Non-hypertensive	37	309	1	1
	Hypertensive	10	27	3.093 (1.387–6.895)	0.97 (0.333–2.826)

*AOR* adjusted odds ratio, *BMI* body mass index, *CI* confidence interval, *COR* crude odds ratio, *DBP* diastolic blood pressure, *DM* diabetes mellitus, *FHDM* family history of diabetes mellitus, *IFG* impaired fasting glucose, *SBP* systolic blood pressure, *SH* smoking habit *TC* total cholesterol, *TG* triglycerides, *WC* waist circumference

* Marginal significance (p = 0.057)

** p < 0.05

*** p < 0.01

The multivariate logistic regression analysis showed that BMI, SBP, and TG were independently associated with IFG. Table 3 below shows that the odds of IFG was 4.26 times higher among overweight subjects compared with underweight subjects (AOR = 4.257, 95% CI 1.345–13.476). Obese individuals were 5.26 times more likely to be prediabetic than underweight individuals (AOR = 5.26, 95% CI 1.138–24.316). Individuals with systolic hypertension were 3.86 times more likely to have IFG as compared to the non-hypertensive individuals (AOR = 3.858, 95% CI 1.62–9.189). Study subjects with hypertriglyceridemia showed higher odds (2.83) of IFG than individuals who had normal triglycerides level (AOR = 2.83, 95% CI 1.451–5.521). Furthermore, a positive family history of diabetes also showed a marginal association with IFG (p = 0.057).

Fisher’s exact test was used to measure the association between undiagnosed DM and risk factors because the cases were few. Therefore, an undiagnosed DM was significantly associated with male sex and hypertriglyceridemia (p = 0.012 and 0.030), respectively.

## Discussion

In this study, around one in eight adults (20 years and above) had impaired fasting glucose (IFG) [12% (95% CI 9–16)], a condition that increases the risk for diabetes. This result is slightly lower than a population based cross-sectional survey done in another sub-Saharan African country, Uganda (20%) [16]. This disagreement might be due to the age difference in the study population; we included adults 20–90 years of age with 43.76 ± 17.29 mean age, while the Ugandan study consisted of an older population of 35–60 years. Moreover, our finding was slightly lower than that of a study conducted in Iran (16.8%) [17] for perhaps, the same reason and owing to the fact that the Iranian study involved both rural and urban dwellers. Our result was similar to that of a study carried out in Poland (9.5%) [18].

In the current study, the prevalence of IFG was not associated with sex which is in line with another study in Brazil [19]. Studies in South Africa [20], Mauritius [21], India [22], and USA [23] confirmed that IFG was higher in males than females. To the contrary, a study in Saudi Arabia [24] presented an opposite finding. These discrepancies need further investigation to discover the underling etiology. The multivariate analysis showed that age was not associated with IFG, while the bivariate analysis showed the opposite. Small sample size might have its own effect. Unlike our finding, several studies reported that the prevalence of IFG had a positive association with older age [25–28].

Regarding our finding, overweight, obesity, hypertriglyceridemia and systolic hypertension were associated with IFG. Positive family history of diabetes also showed a marginal association. Therefore, overweight and obese individuals were risk groups for the development of IFG which was consistent with findings reported by other community-based studies [16, 20, 22, 27]. It has already been a confirmed fact that obesity causes insulin resistance (affect insulin action), and decreases insulin-stimulated glucose disposal, leading to the development of IFG and diabetes mellitus consecutively. Hypertriglyceridemia has a similar scenario with obesity because its blood level is directly related to total body fat accumulation [29]. Moreover, our result demonstrated that IFG had a positive association with systolic hypertension which is confirmed somewhere else [20, 22, 27]. The other established fact is that insulin resistance is a common pathophysiologic feature of obesity, glucose intolerance and, hypertension [30].

The prevalence of undiagnosed DM was 2.3% (95% CI 1.1−4) in this study. This prevalence is comparable with that of Iran (3.85%) [17] and USA (2.8%) [23]. Another study in northwest Ethiopia confirmed that the proportion of previously undiagnosed DM cases was high in both urban and rural settings, while the rate was much higher in rural population. This finding may reflect the low awareness of the community, the public, and primary health care providers about DM [31]. In addition, studies in sub-Saharan Africa identified that more than 40% of the DM cases were previously undiagnosed [3]. Even though the low number of cases limited our analysis of associated risk factor determination, Fisher’s exact test demonstrated that male sex (p = 0.012) and hypertriglyceridemia (p = 0.03) showed a positive association with undiagnosed DM. Other studies also strengthen this finding because of the evidences described under IFG in the paragraphs above [20, 22, 23, 27].

In our study, exclusion of known patients with diabetes mellitus may have affected the prevalence estimate of diabetes. Greater awareness of the age related nature of diabetes may have lead to more recognition of the disorder in these age groups, leaving fewer at risk individuals to be detected in this study. In addition, small sample size and more of younger study population may lower the diabetes estimate.

## Limitations of this study

Primarily, this study did not include patients with diagnosed diabetes mellitus. Therefore, undiagnosed diabetes mellitus alone could not show the exact magnitude of diabetes mellitus in the community. Secondly, some essential factors like diet and physical activity were not assessed because it was not possible to standardize the data obtained from study participants. Thirdly, in comparison to other similar studies, the number of study participants was low (because of budget shortage we were unable to include more participants) which may under or over estimate the prevalence of IFG and undiagnosed diabetes mellitus. Furthermore, this is a study in one rural town and therefore not representative of rural Ethiopia at large, where as it could be used as baseline for further studies with large geographical area.

## Conclusion

The prevalence of IFG was found to be high among adults (age ≥ 20) in Koladiba town. Factors like, overweight, obesity, hypertriglyceridemia, systolic hypertension, and positive family history of diabetes were found to be significantly associated with IFG. Hypertriglyceridemia also showed a positive association with undiagnosed diabetes mellitus. We recommend that awareness should be created regarding healthy life style, early diagnosis and treatment of prediabetes and diabetes. In addition, fasting plasma glucose value is recommended for screening IFG and diabetes currently because it is quick, easy to determine, and acceptable to patients in clinical settings. Therefore, it is advisable to visit health facilities periodically for health checkup including diabetes mellitus.

## Abbreviations

AOR: adjusted odds ratios
BMI: body mass index
CVD: cardiovascular disease
TC: total cholesterol
CI: confidence interval
COR: crude odds ratio
DBP: diastolic blood pressure
DM: diabetes mellitus
IFG: impaired fasting glucose
SPSS: statistical package for social sciences
SBP: systolic blood pressure
TG: triglycerides
WC: waist circumference
